# Root Resective Procedures: A Case Series of Tooth Hemisection and Bicuspidization with Prosthetic Rehabilitation in Contemporary Dental Practice

**DOI:** 10.3390/dj14020122

**Published:** 2026-02-20

**Authors:** Sofia Sokratous, Andreas Krokidis, Nikolaos P. Kerezoudis

**Affiliations:** Dental School, National and Kapodistrian University of Athens, Thivon 2, 11527 Athens, Greece; socratous.sophia@outlook.com (S.S.); nkerez@dent.uoa.gr (N.P.K.)

**Keywords:** endodontic surgery, tooth resection, hemisection, bicuspidization, furcation defects, tooth fractures, rehabilitation, case report

## Abstract

**Background/Objective**: Root resective procedures are well established tooth-preserving techniques used when pathology is confined to one root of a multirooted tooth or in the furcation area. Although in recent years implant therapy has become a standard approach in many cases, the rising incidence of peri-implantitis has renewed interest in classical conservative treatment alternatives, such as hemisection, root resection and bicuspidization. The aim of this study is to present clinical cases in which hemisection and bicuspidization were performed to maintain compromised molars in function and achieve long-term outcomes. **Methods:** This retrospective case series study was conducted in a private dental practice and included three patients treated between 2009 and 2017. The presented cases involved molar teeth exhibiting a vertical fracture or extensive subgingival carries in one root while the remaining root(s) demonstrated favourable periodontal, endodontic and restorative prognosis. An interdisciplinary approach was followed in each case, involving comprehensive clinical and radiographic evaluation including cone beam computed tomography when indicated. The clinical treatment included an endodontic approach (primary treatment or retreatment if required) followed by hemisection or bicuspidization and placement of a permanent prosthetic rehabilitation with full-coverage restoration designed to optimize proper load distribution. Clinical and radiographic follow-up examination was done up to six years in case one, after six months in case two and up to six years in case three. **Results:** The teeth remained in function through their respective follow-up periods. Clinical and radiographic assessments, according to predefined success criteria, demonstrated periodontal stability (probing depth ≤ 4 mm), no evidence of secondary caries or root fracture, absence of clinical symptoms, normal tooth mobility and masticatory function, absence or reduction in periradicular radiolucency, and stable bone levels. **Conclusions:** Resective techniques require an interdisciplinary approach, namely, careful case selection, lege artis endodontic treatment, precise surgical technique, and appropriate prosthetic rehabilitation, in order to provide predictable and long-term outcomes. Within the limitations of this case series, resective techniques appeared to be a reliable and predictable alternative to extraction and implant placement in carefully selected clinical cases.

## 1. Introduction

For over a century, root resection has been used as a treatment approach. This technique was initially introduced by Farrar in 1884 and subsequently adopted and developed by many other clinicians [1]. Today, there is a distinction between the procedure definitions included in the resective techniques. These definitions are as follows: Root separation involves the sectioning of the root complex and the maintenance of all roots [2]. Root resection involves the sectioning and removal of one or two roots of a multirooted tooth [2,3]. Bicuspidization refers to sectioning a mandibular molar and treating the two sections like two premolars [4]. Hemisection is defined as the removal of half of a tooth performed by sectioning the tooth and removing one root, and is frequently used with reference to lower molars [3,4].

These techniques are treatment approaches intended to extend the survival of compromised molars [5,6,7,8]. The main indications for resective root therapy include endodontic, periodontal, restorative considerations and combined endo-perio lesions, primarily of periodontic origin, which cannot be treated otherwise [7,8,9,10,11,12,13,14,15]. Appendix A, Table A1 presents a detailed overview of the indications [3,5,7,8,9,10,11,12,13,14,15,16].

In the past several decades, many studies have demonstrated successful outcomes in preserving teeth with root resection and hemisection [5,6,7,10,17,18,19,20,21,22,23,24]. However, the success of these procedures depends on multiple prognostic factors, including the amount of remaining bone support, the position of the tooth within the arch, the presence of parafunctional habits, and the overall periodontal status [2,5,8,13,20].

Long-term clinical success is achieved through the application of precise surgical techniques, followed by appropriate prosthetic rehabilitation [8,13,20,21,25,26]. Prosthetic management of teeth treated with resective techniques is pivotal to their longevity and success. The use of full-coverage crowns, single or splinted, aids in the even distribution of occlusal forces, protects the remaining tooth structure and enhances oral hygiene. Proper restoration minimizes the risk of fracture and the occurrence of secondary caries as well as the incidence of periodontal disease due to plaque accumulation in the surgically modified area of the root [7,8,13,20,21].

Nowadays, tooth extraction followed by dental implant placement has often become the preferred and chosen treatment in many cases [27]. However, the high incidence of peri-implant complications is an issue and managing these complications has renewed interest in resective therapy as an alternative [6,10,28,29,30,31,32,33,34].

The present case series concerns compromised molars that were preserved for long time by performing resective methods such as hemisection or bicuspidization in a private dental practice.

The cases present clinically relevant scenarios in which pathology was confined to one root of a mandibular molar, while the remaining root(s) demonstrated sufficient periodontal support and restorative potential. In addition, these three cases illustrate different indications, including advanced periodontal involvement, vertical root fracture, subgingival caries and various prosthetic rehabilitation strategies, with extended follow-up periods. The purpose of this case series is to demonstrate that decision-making processes, surgical approach and functional outcomes are achievable with hemisection and bicuspidization in a modern private dental practise.

Written informed consent was obtained from all patients prior to inclusion in the study. Patients were included in this case series if they required the specific dental treatment described, were adults, were able to provide written informed consent and had complete clinical and radiographic documentation. Patients with systemic diseases or medical conditions that could influence treatment outcomes, as well as patients receiving medication affecting their health, healing individuals with incomplete clinical records or follow-up data, and patients who declined or were unable to provide informed consent, were excluded from the study. To protect patient confidentiality while allowing traceability in the clinical database, each patient was assigned a unique static code combining the initial of their name with their patient registry number.

This case series was prepared in accordance with the PRICE 2020 guidelines [35].

## 2. Materials and Methods (Retrospective Case Series)

### 2.1. Case Selection

The cases included in this retrospective case series were identified through a review of the clinical records of patients treated in a private dental practise in Athens, Greece. Patient files from 2009 to 2021 were screened to identify molars that had undergone root resective procedures such as hemisection or bicuspidization as part of their definitive treatment.

The three clinical cases were selected based on strict clinical, radiographic and restorative criteria. All cases involved mandibular molars in which pathology was localized to a single root, including vertical root fracture, advanced localized periodontal destruction or severe subgingival carries, while the remaining roots exhibited sufficient periodontal support and favourable anatomy to allow retention. Comprehensive clinical examination, periodontal probing, periapical radiography and CBCT imaging when indicated were used to confirm the diagnosis and to exclude generalized periodontal disease, extensive bone loss affecting multiple roots or an unfavourable crown-to-root ratio after resection. Only patients with non-contributory medical histories, good oral hygiene, stable periodontal condition after initial therapy and willingness to adhere to long-term maintenance were included. In all selected cases, the retained roots were considered restorable and capable of supporting definitive prosthetic rehabilitation with controlled occlusal loading. These criteria ensure that hemisection or root resection was chosen as a conservative and predictable alternative to extraction and implant placement.

### 2.2. Clinical Techniques

The clinical procedures performed in the cases included in this paper were performed by a general practitioner (S.S.), an endodontist (N.P.K.), and an endodontist also trained in restorative dentistry (A.K.) and followed the clinical protocol described below:

Initial clinical examination and diagnosis were performed by the principal clinician (N.P.K); periodontal charting and initial periodontal therapy were carried out in the first two cases until a stable periodontal status was established, with the exception of the affected root area.

Endodontic treatment was performed by the same endodontist in all three cases (N.P.K.), under operating microscope magnification after rubber dam application and using Ni-TI rotary instruments (Race, FKG, La Chaux-de-Fonds, Switzerland) for root canal preparation under copious irrigation of 2.5% NaOCl solution. Root canal filling was carried out using the System-B apparatus method followed by backfilling with thermoplasticized gutta-percha using the Obtura system, in combination with an AH26 silver-free sealer (Dentsply, NC, USA). A postoperative periapical radiograph was obtained to verify the quality of the obturation.

Prosthetic rehabilitation was conducted by A.K. in all cases; core build-up was done with metal or fibre posts when needed, and an impression was taken using the simultaneous double-mixing technique with heavy-body and light-body silicone (Express STD putty and Express light-body, 3M ESPE, MN, USA) after gingival retraction using 000 and 00 cords. Preparation margins were placed supragingivally or equigingivally in order to preserve the biological width. The remaining teeth were restored with a full-coverage crown either with a mesial wing, splinted or as an abutment. Special attention was given to the occlusal plate designed to eliminate lateral extrusive forces, direct masticatory forces along the axis of the root and ensure optimal marginal adaptation.

### 2.3. Follow-Up

Clinical assessment included an evaluation of symptoms, gingival swelling, presence of sinus tracts, periodontal charting, tooth mobility, and recovery of masticatory function. Radiographic evaluation using the paralleling technique assessed the periradicular status.

Treatment success was defined by the absence of clinical symptoms, lack of sinus tract, periodontal probing depth ≤ 4 mm, absence of bleeding on probing (BoP), normal tooth mobility or not progressively increased mobility, while the masticatory function was normal without any signs of discomfort. Percussion of the tooth or palpation examination was not sensitive, while radiographic examination of the resected tooth revealed the absence or reduction in periradicular radiolucency, absence of recurrence of caries, that the bone level was stable, and no evidence of root fracture was noticed.

Treatment failure was defined as the persistence of clinical symptoms, sinus tract establishment, increased periodontal probing depth >4 mm, bleeding on probing, gradually increased tooth mobility, unchanged or enlarged periradicular radiolucency, secondary caries, further bone loss or evidence of root fracture.

### 2.4. Case Reports

#### 2.4.1. Clinical Case No. 1

A 45-year-old male patient, non-smoker with non-contributory medical history, came to the dental clinic in 2009 complaining about a slight pain during eating at the right side of his mouth.

Diagnosis:

Clinical examination confirmed a localized gingival inflammation on the lower right side of the mouth adjacent to teeth #46 and #47, despite the good oral hygiene conditions of the patient. A 10 mm deep, narrow periodontal pocket was evidenced in the mesial root of the lower right second molar (#47) upon clinical examination with BoP. Mobility and furcation involvement were not present. The first molar #46 exhibited a 6 mm periodontal pocket with BoP only on the distal root, while the mesial presented a 4 mm without BoP and grade I furcation involvement. Mobility was not present.

**Figure 1:**
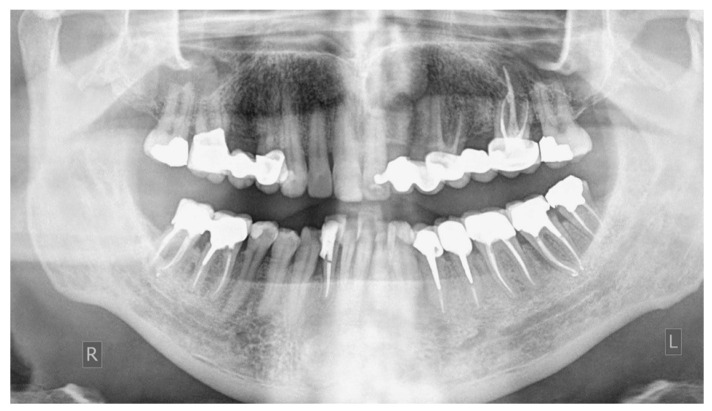
Initial OPT X-ray.

From radiographic examination with OPT (Figure 1), moderate periodontal bone loss, on the lower right side of the patient, was observed (coronal third of the roots).

**Figure 2:**
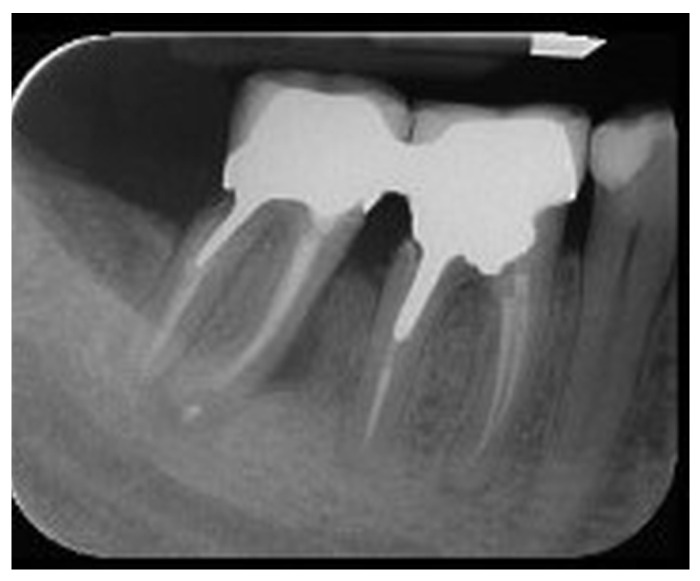
Periapical X-ray of #46, #47.

A periapical radiograph in the molar region of teeth #46–47 revealed that there was a radiolucent area in the middle third of the mesial root of tooth #47. This lesion was located mesially and it seemed to be rather well defined in this area but coronally the bone loss had a J-shape appearance. In addition, subgingival carries in the distal roots of #47 and #46 were also detected (Figure 2). Both teeth had been restored with cast posts and splint fused to metal ceramic crowns. The crestal bone between the two teeth appeared to be moderately resorbed on the coronal third of the root.

These combined clinical and the radiographic findings are characteristic of vertical root fracture of the mesial root of tooth #47 [36]. The prognosis of both teeth was evaluated as unfavourable due to the presence of subgingival caries, the extensive restorations, the minimal remaining tooth structure and the vertical root fracture of the mesial root of #47.

Treatment planning:

After informing the patient with these findings, treatment options were presented. Tooth #47 had to be extracted, while the prognosis of tooth #46 was poor due to an extensive carious lesion, rendering it non-restorable. Thus, the first treatment option was extraction of both teeth #47 and #46 and placement of two implants supporting fixed crowns, and the second treatment option was a more conservative approach, namely extraction of tooth #47, hemisection and extraction of distal root of #46 and after healing, placement of an implant-supported crown in the area of #47, retreatment of the mesial root of #46, build-up of the remaining mesial root and a restoration with a zirconia crown.

The patient expressed his scepticism about implant therapy and he preferred to keep his natural teeth; therefore, he decided to proceed with the second, more conservative treatment option.

Treatment:

Initial periodontal therapy was performed first, and the patient was given oral hygiene instructions. After achieving a stable and acceptable periodontal status, endodontic retreatment of the mesial root of #46 was performed and extractions of the right second lower molar and the distal root of the first lower molar followed, and no attempt was made for socket preservation since the buccal and lingual bone plates were intact. A few days later, the retained mesial root was built-up with Clearfil DC core plus resin (Kuraray Noritake, Tokyo, Japan) and fibreglass post and, finally, a zirconia crown restoration was inserted and bonded with Panavia SA (Kuraray Noritake, Japan) after 4 weeks. The patient was informed that the post-extraction site must be left to heal for at least 5–6 months before placing an implant in the region of the missing tooth #47 and the distal root of #46. However, the patient did not return to the office for implant placement in due time despite recall notifications.

Subsequent visit:

Five years later (2014), the patient appeared to the dental office, complaining about a pain occurring upon chewing in the lower left side.

Diagnosis:

The clinical examination in this area revealed that the gingiva in the molar region was moderately inflamed, percussion of the molars elicited a slight sensitive reaction on tooth #37, there was grade I furcation involvement and no mobility was present. However, a deep narrow pocket (12 mm) in the buccal aspect of the mesial root of the second molar (#37) was found upon probing, accompanied by BoP. The distal root appeared to have moderate bone loss located on the coronal third of the root, no pocket depth > 4 mm and no BoP was evident.

**Figure 3:**
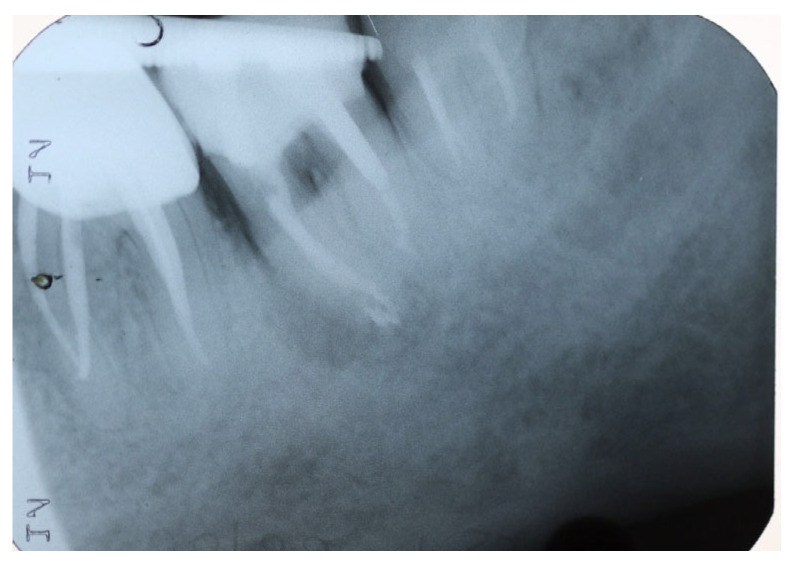
Periapical X-ray of tooth #37 showing a J-shaped radiolucent area at the mesial root.

A periapical radiograph in this area showed a J-shaped radiolucent area in the mesial root of tooth #37 extending from crest of the alveolar ridge to the apex of the tooth (Figure 3).

**Figure 4:**
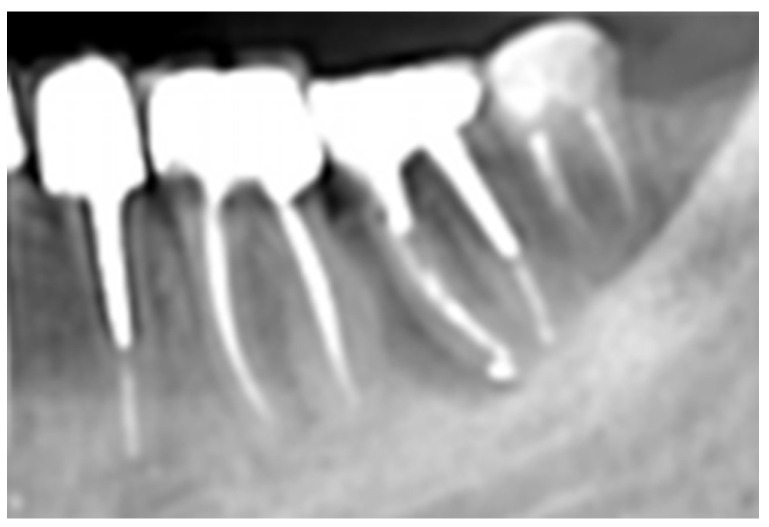
CBCT section of tooth #37 illustrating the pattern of bone resorption alongside the mesial root.

**Figure 5:**
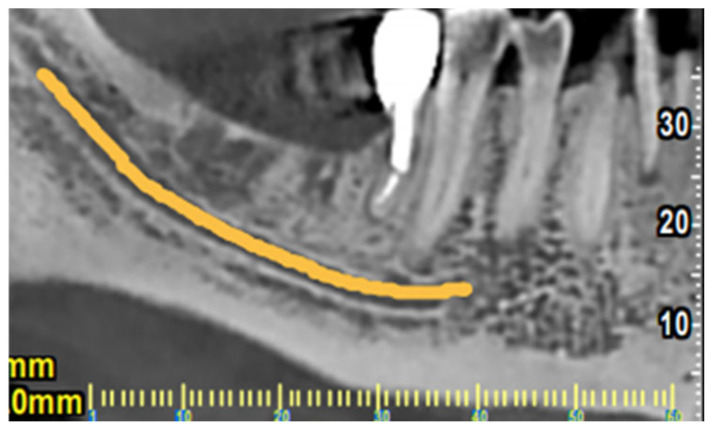
CBCT view of the lower right region of the mandible used to evaluate bone dimensions, bone quality, and the course of anatomical elements for implant placement (e.g., inferior alveolar nerve marked with orange). The retained mesial root of #46 remained functional after hemisection and crown restoration without signs of periapical pathology, root fracture or caries.

Based on these clinical and radiographic findings, a CBCT examination of the lower jaw was suggested to evaluate firstly if there was a vertical fracture on the mesial root of tooth #37 and secondly to verify the consistency and dimensions of the bone for implant placement in the region of #46–#47 (Figure 4 and Figure 5). From the CBCT evaluation, the suspected vertical root fracture in the mesial root was confirmed [36] (Figure 4).

Treatment planning and treatment:

According to these findings, the following treatment plan was proposed: (a) placement of an implant in the region of the missing second molar in the right side as planned five years before and (b) resection of the mesial root of #37 followed by a bridge restoration supported on tooth #36 to the distal root of #37 and the mesial root of #37 as a pontic.

**Figure 6:**
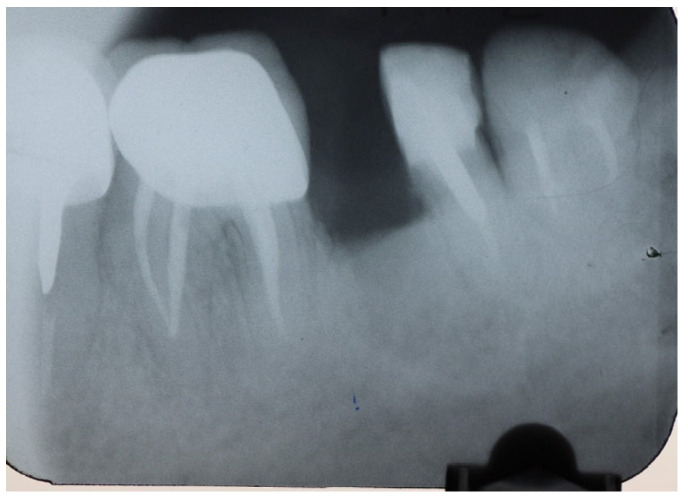
Postsurgical X-ray following hemisection of tooth #37.

Initially, a periodontal recall treatment that included plaque control, tooth scaling and light curettage, where necessary, was performed. Since endodontic treatments of the teeth were acceptable without any periapical lesion and no caries were detected after removing the crowns, it was decided to proceed with the proposed treatment plan without retreatment. Hemisection was performed using the vertical cut method, without flap elevation. The vertical cut was made with a long diamond bur to divide the two roots and then the mesial portion of the tooth, including the mesial root and the equivalent portion of the crown, were extracted with premolar forceps [4,12,13]. A postsurgical radiograph was taken to verify that no undercuts and ledges were left [13,26] (Figure 6). No attempt for socket preservation was made and the extraction site was left to heal normally.

The patient was advised to return after 20 days for the placement of prosthetic reconstruction [10]. After healing of the extraction socket, a prosthetic rehabilitation (porcelain fused to metal bridge) was manufactured, fitted and bonded with Ketac Cem Plus Automix (3M ESPE, MN, USA). Special care was given to minimize the occlusal forces, especially on the distal root of tooth #37.

As scheduled, an appointment for implant placement in the area of #47 was planned afterwards.

Follow-up and outcomes:

A follow-up appointment 1 year after (2016) was planned. During this recall appointment, the clinical examination of teeth #36 and #37 revealed the absence of clinical pathological findings, the prosthetic restoration was functional, no pockets were found upon probing, and no BoP and no evidence of root fracture or caries were seen.

**Figure 7:**
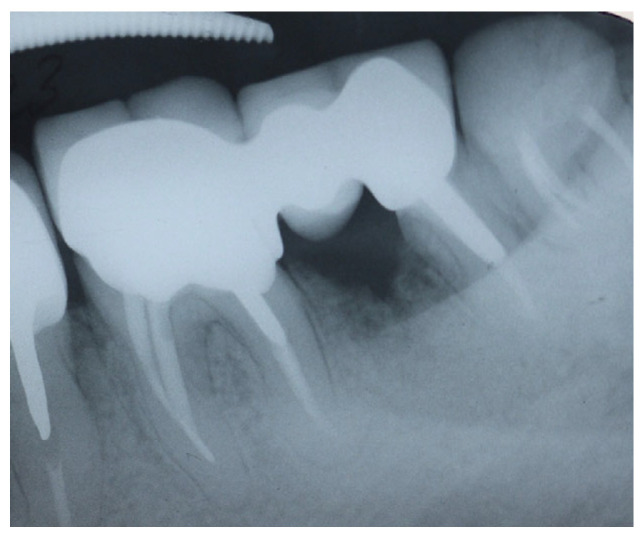
X-ray at 1-year recall, after restoration. An ongoing bone formation on the extraction socket is evident.

An X-ray taken of teeth #36 and #37 revealed a radiographic bone formation in the extraction socket and stable and healthy periodontal and periapical tissues (Figure 7).

**Figure 8:**
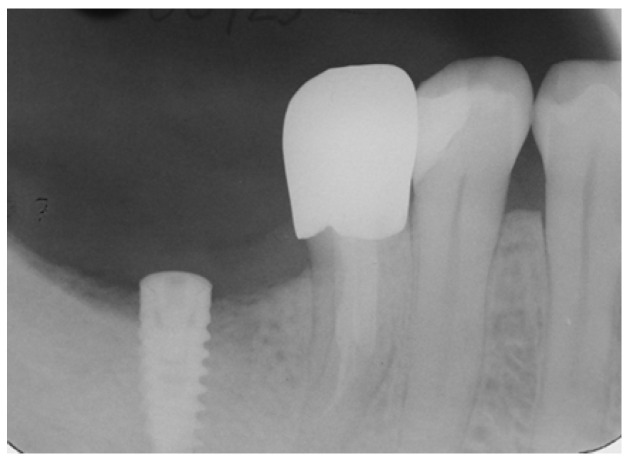
Periapical X-ray after implant placement showing osseointegration and the mesial root of #46 with normal bone tissues and absence of periapical pathology.

On the other side, an X-ray of the lower right region demonstrated that the implant placement showed osteointegration while the retained mesial root of #46 showed stable bone levels, the absence of periapical pathology and no evidence of root fracture or caries (Figure 8).

**Figure 9:**
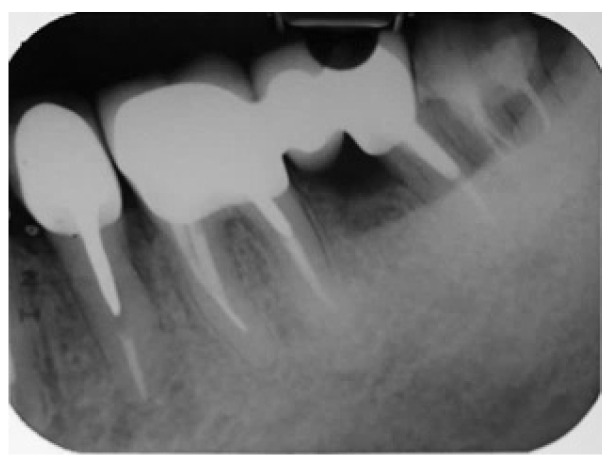
Five years follow-up periapical X-ray. Stable crestal bone level and radiographic bone fill in the previously extracted area.

**Figure 10:**
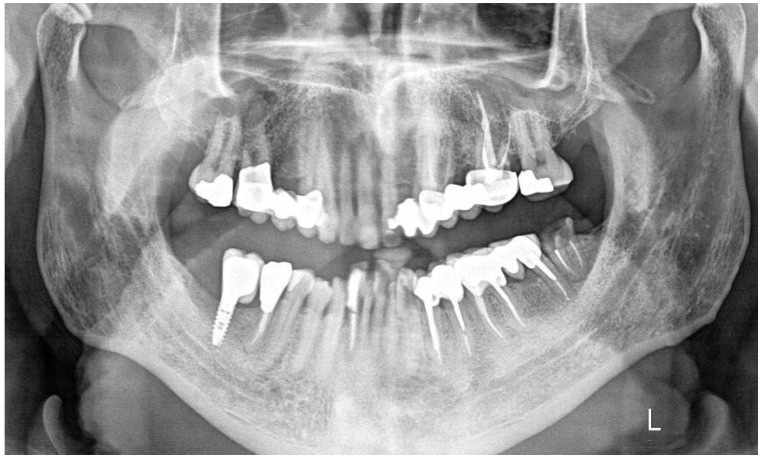
OPT at 5-year recall for teeth #36-#37, implant on #47 and 12 years after crown cementation on tooth #46. Absence of periapical pathology, stable crestal bone level, no evidence of caries or fracture consistent, with stable clinical function.

Follow-up examination five years after completion of the treatment (2021) (Figure 9 and Figure 10) revealed that both right and left sides were functioning and healed properly.

Clinical examination confirmed the absence of periodontal pockets, BoP and secondary caries.

Radiographic examination demonstrated the absence of periapical pathology, progressive bone loss and caries and no signs of root fracture.

#### 2.4.2. Clinical Case No. 2

**Figure 11:**
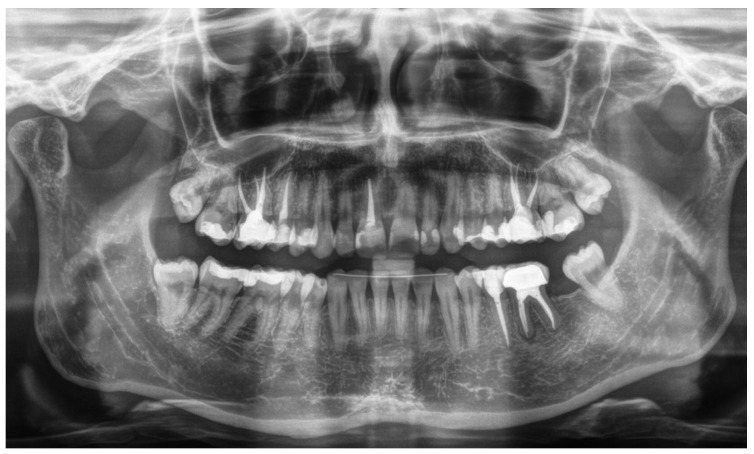
OPT of the patient with evident lesion in the mesial root of #36.

**Figure 12:**
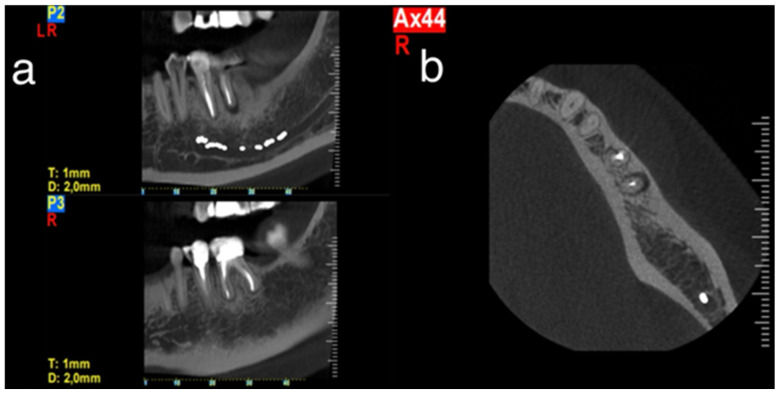
CBCT images of tooth #36 confirming localized pathology of the mesial root. (**a**) Coronal view, radiolucency around the mesial root of tooth #36. (**b**) Axial view, a very localized, angular, fissure-like radiolucency on the buccal surface of the mesial root of tooth #36.

A 50-year-old female patient, non-smoker with non-contributory medical history, was referred to our clinic in 2017 complaining of a recent growing pain and discomfort in the lower left posterior region. Clinical examination revealed a deep and narrow 12 mm periodontal pocket around the mesial root of tooth #36, BoP, and moderate local inflammation on the buccal gingiva, with overall good oral hygiene and low plaque index (<25%). No mobility or furcation involvement were present. Initial radiographic examination showed a periapical radiolucency associated with the mesial root of endodontically treated tooth #36 (Figure 11). Based on patient symptoms and the clinical findings, a possible diagnosis of vertical root fracture of the mesial root was established, and the patient was referred for a CBCT examination to confirm the diagnosis if possible. CBCT demonstrated a localized lesion extending along the buccal surface of the mesial root, confirming the presence of a vertical root fracture in this root (Figure 12).

The treatment options presented to the patient included: (a) extraction of the tooth and placement of an implant (b) extraction followed by bridge restoration of the region using teeth #35 and #38 as abutments and (c) hemisection of #36 and a crown restoration. For the patient, the placement of an implant was not an option due to personal preferences. The bridge restoration between #35 and #38 presented some technical difficulties since the tooth #38 was mesially tilted due to the fact that there was no restoration after the early extraction of tooth #37. This required more invasive preparation of the mesial aspect of tooth #38 in order to achieve parallel longitudinal axes of the abutments, thereby increasing the risk of irreversible pulpitis and the need for endodontic treatment. In addition, for a proper restoration placement, a gingivectomy around tooth #38 was needed for crown lengthening. The patient considered this scenario as an overtreatment. So, the only remaining option was to maintain as much as possible of the substance of tooth #36 and try to restore it in the most minimal and long-lasting way possible. Having this in mind and for better masticatory load distribution and stability [8,20,37,38], the alternative of a bridge between #35 and distal root #36 was proposed to the patient but she refused to proceed in this option. In order to align with the patient’s requests and produce a stable and long-lasting restoration, a solution of restoring the distal root of tooth #36 with a crown having a mesial wing extension was selected.

The decision to restore the retained distal root of tooth #36 with a single crown incorporating a mesial wing bonded to #35 was based on biomechanical and patient-related considerations. The missile wing design was selected as a minimally invasive compromise intended to enhance lateral stability and reduce the risk of overload and eventual fracture of the retained root, while preserving tooth structure and maintaining conditions that enhance effective oral hygiene.

However, this approach possesses possible risks, including potential debonding of the wing, increased danger for caries, and altered load transfer to the abutment tooth (#35). To minimize these risks, the wing was designed with a passive fit, bonded to enamel within a well-defined occlusion notch and strict control over occlusal contacts was established to eliminate non-axial forces. These modifications, in combination with effective oral hygiene from the patient, make this approach a clinically acceptable alternative.

**Figure 13:**
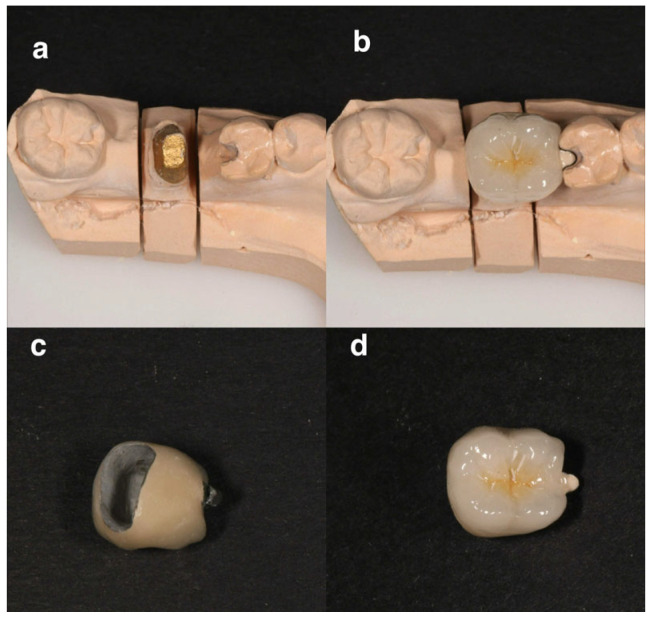
Dental crown of #36 design and placement on the dental cast. (**a**) Occlusal view of the dental cast, (**b**) dental crown placed on the cast, (**c**) mucosal view of the dental crown with mesial wing, (**d**) occlusal view of the crown.

The treatment proceeded with hemisection of the first molar (#36); a full-thickness mucoperiosteal flap was elevated and since the fracture was visible buccally and hemisection was performed using the vertical cut method, with a long-fissured diamond bur. The mesial root along with the equivalent part of the crown was extracted using premolar forceps. Sutures were placed using 4-0 vicryl sutures. The patient was given verbal and written postoperative instructions. After 10 days, a recall appointment was scheduled. At the recall appointment, normal socket and soft tissue healing was noted and the sutures were removed. Fifteen days after suture removal, the patient returned to the office, the remaining abutment was shaped, and a provisional crown was manufactured chairside and cemented until proper healing of the socket occurred. When healing was completed, preparation of the remaining tooth crown was finished, a special notch on distal occlusal surface on tooth #35 was made, an impression was done, and a porcelain crown fused to metal having a mesial wing was manufactured. The mesial metal wing was designed to be bonded on the prepared notch on tooth #35 (Figure 13). Final restoration was made with cement using a Ketac Cem Plus Automix (3M ESPE, MN, USA).

Six months after, at the recall appointment, clinical and radiographic examinations of teeth #35 and #36 were conducted. Clinical examination confirmed the absence of periodontal pockets and BoP of both tooth #35 and #36. No evidence of root fracture or secondary caries was detected. Also, the low plaque index (<25%) found confirmed the good oral hygiene of the patient.

**Figure 14:**
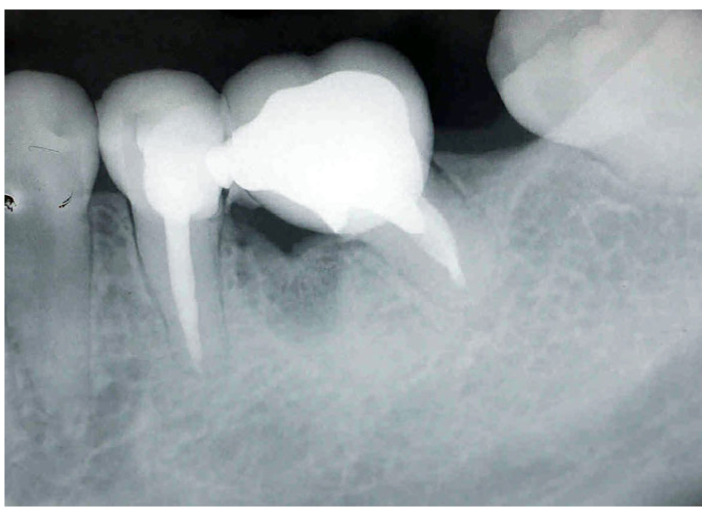
Six-month follow-up, after cementation of the crown.

Radiographic examination demonstrated the absence of periapical pathology and no evidence of progressive bone loss, caries or root fracture. The radiographic findings are consistent with normal healing of the extraction socket (Figure 14).

#### 2.4.3. Clinical Case No. 3

**Figure 15:**
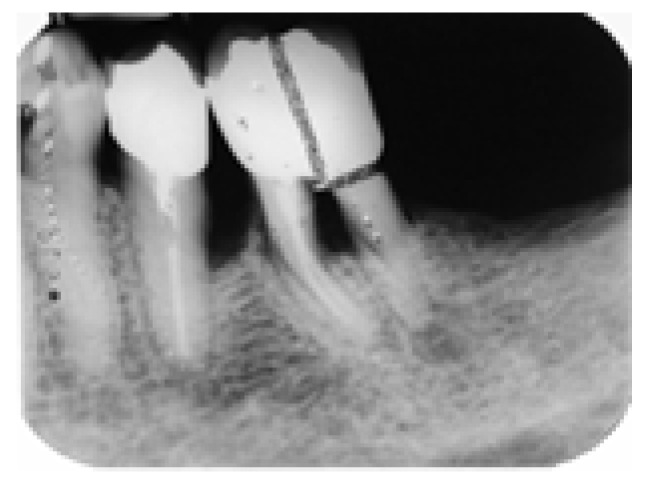
Initial periapical X-ray of tooth #36.

A 55-year-old female patient, with no contributory medical history and a non-smoker, was referred by a periodontologist to our clinic for endodontic evaluation and, if possible, retreatment of the mesial root of the lower left first molar. According to the referral records and as was indicated in the accompanying periapical radiograph (Figure 15), the initial treatment plan included removal of the distal root of tooth #36 by hemisection. Study of the periapical radiograph showed a furcal involvement in tooth #36 and a periapical lesion of the untreated distal root. Clinical investigation revealed a type III furcation involvement, first grade mobility combined with a deep periodontal pocket 7 mm in the furcation area and BoP, while the presence of initial caries was detected at the distal margins of the porcelain fused to metal crown. The remaining periodontal support on the mesial root was comparatively favourable, with probing depths not exceeding 4 mm and absence of bleeding when probing.

**Figure 16:**
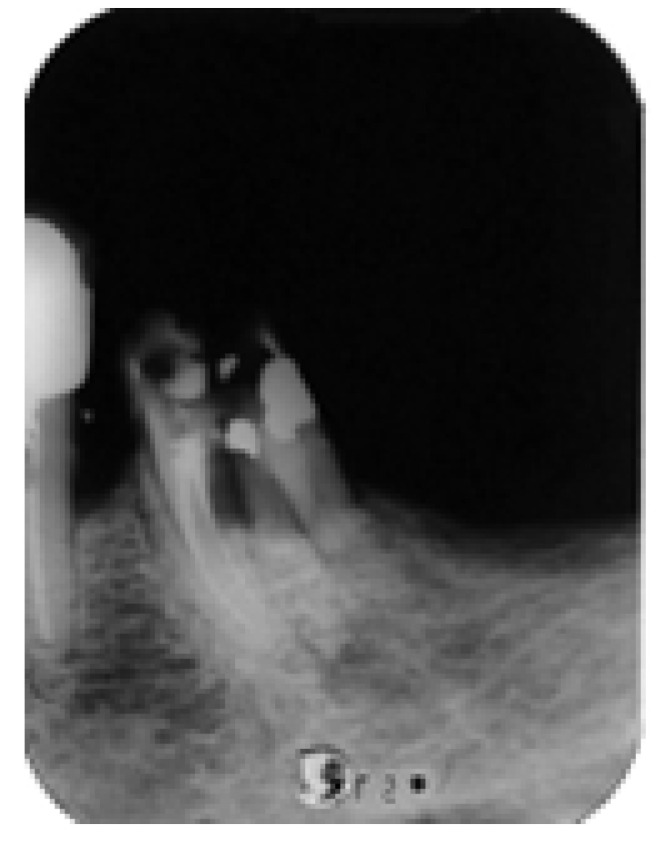
X-ray after crown removal showing the existence of the second distal root of tooth #36.

Based on these findings, the tooth was given a questionable to poor prognosis. The patient was informed about the prognosis of the tooth but insisted on trying to save it, because of a previous experience of failing implants on the lower right region. As mentioned, the patient was in a regular maintenance recall program and in order to examine if the tooth crown was restorable, we decided to proceed with the removal of the crown. As shown in Figure 16, a periapical radiograph taken after removal of the crown revealed the presence of caries in the disto-buccal part of the tooth and the existence of two distal roots having a grade II furcal involvement in between them.

The patient was informed about these findings, and the treatment options were as follows: (a) Extraction of the tooth and an implant placement. This option was immediately excluded by the patient since she had a previous experience of a failing implant placement on the right side of her mouth due to periimplantitis. (b) The second option offered was to first perform the endodontic treatment and then a classical root hemisection only of the distal-buccal root, which had a poor prognosis due to the extensive caries and grade II furcation involvement, and, thereafter, a bicuspidization of the two remaining roots, namely the mesial and distal lingual. Finally, prosthetic rehabilitation was suggested to restore the two separated roots as premolars with splinted crowns. Another alternative was to also replace the crown of tooth #35 and to splint it with the restoration of tooth #36, but this was excluded by the periodontologist, since it would render daily oral hygiene more difficult.

**Figure 17:**
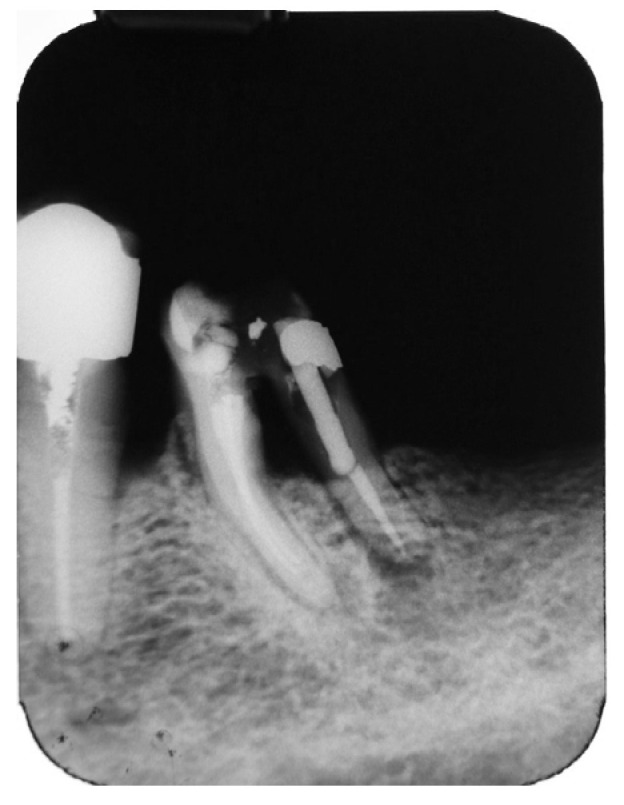
Postsurgical X-ray after the built-up of the tooth and removal of the distal-buccal root of tooth #36.

According to the second treatment plan, we proceeded with endodontic treatment and built-up restoration with composite resin and a prefabricated metal post of the disto-lingual root under dental dam isolation, with a design that allows oral hygiene in the interdental space. This was followed by initial hemisection and extraction of the severely compromised distal-buccal root and then bicuspidization of the mesial and distal-lingual roots (Figure 17).

After socket healing, the patient returned for the final restoration, which consisted of two splinted metal–ceramic crowns in the separated roots. Final restoration was designed with special care to the interdental space to maintain the good oral hygiene and cemented with Ketac Cem Plus Automix (3M ESPE, MN, USA).

The decision to retain tooth #36 was based on an interdisciplinary assessment that considered the patient’s motivation or hygiene capability, anatomical root configuration and the possibility of eliminating the frication defect through resection of the distal-buccal root and bicuspidization of the remaining mesial and distal lingual. Strict periodontal maintenance protocol was established, consisting of professional supportive periodontal therapy, enforcement of our and the periodontist’s hygiene distractions and regular clinical and radiographic monitoring.

**Figure 18:**
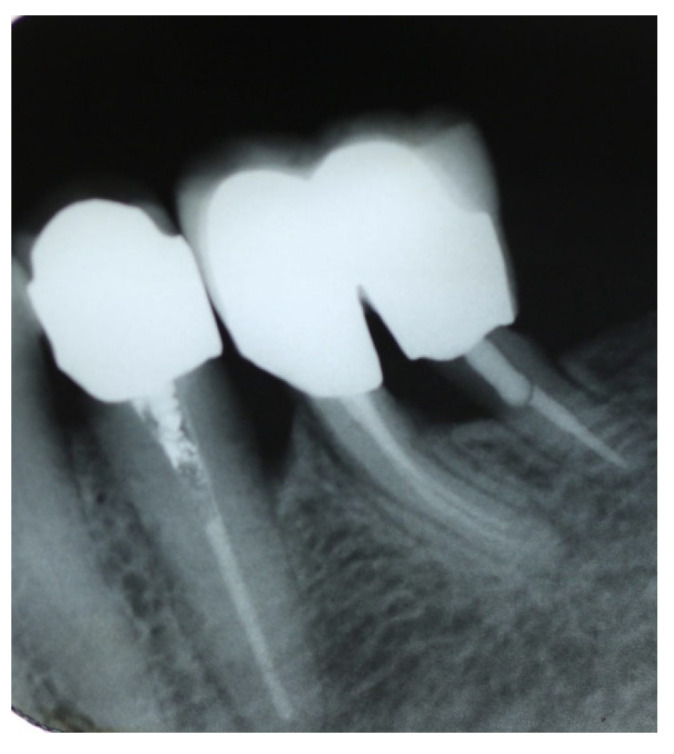
Recall X-ray after 6 months of prosthetic rehabilitation.

A six-month recall examination revealed that the restoration was functioning well and the patient was fully satisfied. Clinical examination revealed no signs of inflammation, absence of clinical symptoms, elimination of the pocket located in the furcation and only slight mobility of the tooth was present. BoP was not evident and the plaque index was low (<25%), which indicated that the patient was able to maintain oral hygiene properly according to instructions. Radiographically, no lesions were evident, and bone levels were stable (Figure 18). No findings of root fracture or secondary caries were found neither clinically nor radiographically.

The patient was advised to keep strictly to the regular periodontal recall examination program.

Recall examination after 6 years showed that the tooth remained functional over the years. Clinical examination confirmed the stable periodontal status of the patient, absence of clinical symptoms, functional prosthetic restoration, and the absence of caries or root fractures. Slight mobility was present, but it was not progressively increased compared to the previous follow-up examination.

**Figure 19:**
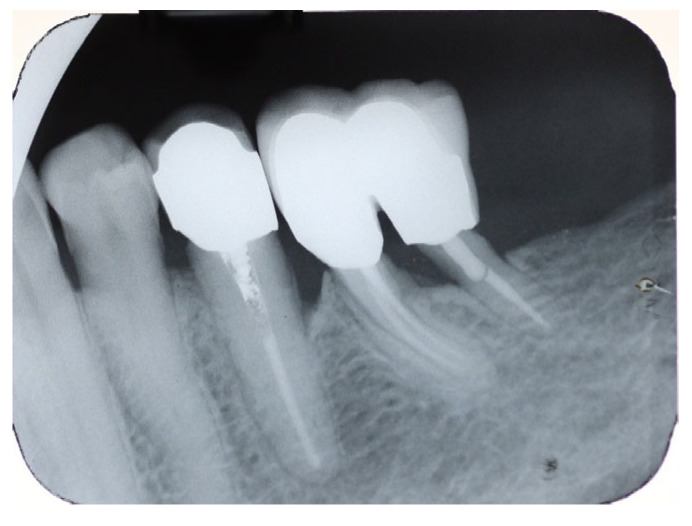
Six-year recall X-ray.

Radiographic evaluation demonstrated normal periapical and periodontal tissues, no progressive bone loss and no evidence of caries or root fracture (Figure 19).

### 2.5. Patient Perspective

All patients expressed satisfaction with the chosen treatment approach and the functional outcome of their restorations. Preservation of their natural teeth was considered a significant benefit, particularly with who were reluctant to undergo implant therapy due to various reasons. During follow-ups, patients reported normal masticatory function and absence of discomfort. None of the patients reported functional limitations or dissatisfaction with the restorations.

## 3. Results

A summary and the results of the three cases are presented in Table A2.

## 4. Discussion

In recent years, the concept of tooth preservation has gained renewed attention as a biologically driven alternative to extraction and implant placement. While implant therapy has been considered the gold standard for tooth replacement in many clinical situations for several decades [39], the incidence of periimplantitis has risen steadily over the years [28,29,30,31,32,33,34,40]. Numerous studies have evaluated resective techniques such as root resection, hemisection and bicuspidization over the past decades, and have reported favourable clinical outcomes when case selection, periodontal stability and restorative planning are appropriately addressed [4,8,20,41]. Recent case reports continue to document the management of compromised molars treated with resective techniques, highlighting the potential of these minimally invasive procedures when combined with a multidisciplinary approach [41,42,43,44,45,46].

The presented clinical cases aimed to illustrate the clinical application of hemisection and bicuspidization in selected mandibular molars with pathology confined to one root, managed through an interdisciplinary approach.

All the treated teeth were maintained in function following hemisection or bicuspidization according to the success criteria, through their respective follow-up periods. These observations suggest that, under favourable local conditions, with careful case selection and coordinated endodontic, periodontal and prosthetic management, root resective procedures, such as hemisection and bicuspidization applied in the present cases, can allow continued function of compromised molars. However, these outcomes reflect individual clinical trajectories and were not assessed using standardized, quantitative outcome measures.

It should be noted that in the presented clinical cases in our study, endodontic treatment or retreatment was completed before the surgical resective procedures. This approach is supported by the literature, which has reported improved predictability and overall success of this sequence of care [2,3,12,47]. However, cases in which resection can be performed on a tooth with a vital pulp (vital root resection), particularly in periodontally compromised multirooted teeth, are also described in the literature [48,49,50]. Several complications have been reported in association with this approach; some of them include difficulty achieving adequate anesthesia due to chronic inflammation, challenges in performing proper endodontic treatment due to altered tooth morphology, potential pulp calcification, internal resorption resulting from chronic inflammation, pulp necrosis and severe postoperative pain [12,13,48]. As a result, this approach remains controversial, as success rates vary widely and it is generally considered a high-risk procedure [49,50]. The prognosis of vital root resection is generally poorer compared to cases where endodontic treatment is performed before the root resection.

From a clinical perspective, prosthetic rehabilitation plays a critical role in long-term function, as even a successful hemisection will eventually lead to tooth loss within a certain period, if not followed by proper prosthetic rehabilitation. The most frequent failures were due to fracture, secondary caries, or recurrent periodontal disease resulting from plaque accumulation in the treated area [51]. In our clinical cases, full-coverage crowns were used to restore the preserved roots, as they improve the distribution and control of occlusal forces, protect the tooth from secondary caries and promote an environment that supports effective oral hygiene [3,4,13,15,38,51]. Special attention was given to the management of parafunctional habits [20] and occlusal stresses to avoid possible fractures [4,11,15,20,47,52].

The decision about whether a crown restoration should be single, part of a prosthetic restoration, or splinted to an adjacent tooth was based on factors including the remaining roots, the surrounding bone support, the tooth position in the arch, and the overall prosthetic treatment plan [4,8,12,21,25]. In the first clinical case, on the lower right side, the mesial root was retained and restored with a full-coverage crown, while an implant was positioned and placed distally, resulting in favourable distribution of the occlusal stresses. In the same clinical case, a bridge on the lower left side was manufactured and there was not a single crown on the distal root, since the literature indicates that when a distal root serves as a last abutment, it is preferable to splint it so as to enhance stability and load distribution forces [12,20,23,25,53]. In the second case, as the patient declined splinting the distal root with a bridge or crowns, an alternative yet effective solution was provided to the patient using an occlusal wing to reduce the risk of root fracture [8,13,20]. Lastly, in the third case, the two divided roots were splinted with two crowns. All three cases were restored with attention to minimize excessive occlusal forces, allowing adequate oral hygiene while taking into consideration patient preferences.

These clinical decisions are also supported by the broader literature. Teeth functioning as terminal abutments demonstrate lower success rates because they are subject to greater occlusal forces [5,20,23,25]; further reduction in the prognosis is observed when there is limited bone support around the retained root [4,7,8]. For this reason, splinting or using the retained root as an intermediate bridge abutment when there is low bone support is generally recommended to improve force distribution and avoid fracture, especially in mandibular molars where fracture represents a common cause of failure [4,5,8,10,20], and, when possible, these teeth should not be used as terminal abutments [8,21].

As mentioned above, the long-term survival and success of root resective procedures have been widely investigated; however, reported outcomes vary considerably depending on study design, duration of follow-up and case selection. Clinical studies indicate that favourable results are generally associated with carefully selected cases and well-designed restorative management. Some of the studies such as Bergenholtz et al., Hamp et al., and Klavan et al. at early investigations, which predominantly included patients treated under favourable periodontal and restorative conditions, reported high success rates of 91–94% on follow-up periods ranging from one to ten years [17,18,19]. Later, Carnevale et al., in a cohort receiving structured periodontal maintenance, reported survival rates approaching 99% at five years of observation and more that 93% at ten years [7]. A progressive decline in survival was noted by Falabella et al., who reported survival from 97.735 to 90.91% at ten years, suggesting cumulative biological and functional challenges [10]. In a retrospective study, Derks et al. found a 90,6% survival rate after 10 years of observation, highlighting acceptable long-term outcomes despite grater variability in case selection and maintenance [24].

In general, these findings suggest that root resection and hemisection can provide satisfactory long-term outcomes in selected cases. Lege artis endodontic treatment, controlled occlusal forces, and stabilization of periodontal support, as well as prosthetic restorations designed to limit biomechanical stress, remain essential components of success. The present case series does not allow comparative conclusions with implant placement but illustrates that hemisection and bicuspidization may contribute to prolonged retention of natural teeth and preservation of the surrounding bone. As observed in the follow-up radiographs of the cases presented above, bone preservation is evident over the years, which may be advantageous if implant placement is required in the future under more favourable biological conditions.

### Limitations

This case series is limited by the small number of cases and the absence of standardized outcome measures, which restrict the generalizability of the findings. Follow-up duration varied between cases and the exploratory nature of the study means that the results should be interpreted as illustrative of the feasibility of resective techniques rather than definitive evidence of their predictability or long-term effectiveness.

## 5. Conclusions

This case series study illustrates the clinical feasibility of resective techniques for the management of compromised molars when a single root is affected and appropriate periodontic, endodontic and restorative conditions are present. The detailed preoperative diagnostic evaluation led to careful case selection and, combined with an interdisciplinary treatment approach, contributed to successful clinical outcomes. Although limited by the small number of cases and non-standardized outcome measures, these examples suggest that the presented techniques may represent a conservative treatment alternative in selected situations, allowing preservation of the natural dentition and supporting future implant placement if needed through maintenance of the alveolar bone. While these cases illustrate the potential of the resective techniques, further research with larger cohorts and standardized outcome measures is needed to evaluate the predictability and long-term results.

## Data Availability

The data presented in this study is available on request from the corresponding author due to privacy or ethical restrictions.

## Abbreviations

The following abbreviations are used in this manuscript:

CBCT	Cone Beam Computed Tomography
OPT	Orthopantomogram
GP	General Practitioner
BoP	Bleeding on Probing

## Appendix A

**Table A1:** Indications for resective treatment.

Periodontal	Endodontic	Restorative	Endo-Perio Lesions
Furcation involvement grade II and III	Root resorption (only in one root)	Subgingival caries (affecting a single root)	Combined endo-perio lesions, (primarily periodontic, affecting a single root)
Significant bone loss (affecting one root)	Vertical root fracture (of one root)	Extensive decay extending into the furcation area	
Extensive gum recession in a root	Iatrogenic damage only one root or the pulp chamber (cannot be managed with periapical surgery due to anatomical limitations)	Severe root exposure (complicates proper prosthetic restoration)	
	Extensive tooth structure loss (e.g., attempt to remove a broken file)		

**Table A2:** Summary and results of the three cases.

	Variable	Case 1	Case 2	Case 3
**Age/Sex**		45/Male	50/Female	55/Female
**Tooth**		#46 (mesial root retained)—2009 #37 (distal root retained)—2014	#36 (distal root retained)	#36 (mesial and distal-lingual roots retained)
**Main indication**		Subgingival caries Vertical root fracture	Vertical root fracture	Furcation involvement grade III and II (between lingual and buccal)
**Clinical findings**	Probing depth (mm)	#47: 6,5,10 5,6,10 #46: 6,5,4 6,6,4 #37: 12,6,4 12,6,3	#36: 12,4,3 12,4,3	#36: 3,7,3 4,7,4
	CAL (mm)	#47: 6,5,10 5,6,10 #46: 6,5,4 6,5,4 #37: 12,6,4 12,6,3	#36: 12,4,3 12,4,3	#36: 5,7,7 5,7,6
	BoP	+ − − + − −	+ − − + − −	− + − − + −
	Mobility	#47: − #46: − #37: −	−	Grade I
	Furcation Involvement	#47: − #46: Grade I #37: Grade I	−	Grade III
**Procedure(s)**		Hemisection	Hemisection	Hemisection of the distal-buccal and bicuspidization of the remaining lingual and mesial roots
**Endodontic status**		Retreatment of the mesial root #46 Adequate endodontic treatment distal root #37	Adequate previous endodontic treatment	Endodontic treatment of the retained roots
**Restoration type**		Single full-coverage zirconia crown (#46) Metal–ceramic bridge #36–37# (distal root)	Full-coverage metal-ceramic crown with mesial wing bonded to #35	2 splinted full-coverage metal–ceramic crowns as premolars.
**Follow-up**		12 years (#46) 5 years (#36 = #37) —2021	6 months	6 years
**Complications**		none	none	slight mobility
**Outcomes**	Clinical symptoms	absence	absence	absence
	Probing depth (mm)	≤4 mm #46: 3,3,3 3,3,3 #37: 3,2,2 3,2,3	≤4 mm #36: 2,2,3 2,3,2	≤4 mm #36 (mesial): 3,3,3 4,3,4 #36 (distal-lingual): 4,4,3 4,3,3
	CAL (mm)	#46: 3,3,3 3,3,3 #37: 3,2,2 3,2,3	#36: 2,2,3 2,3,2	#36 (mesial): 5,5,7 5,6,7 #36 (distal-lingual): 7,5,5 7,5,6
	BoP	absence	absence	absence
	Mobility	normal	normal	slight (+), not progressively increased
	Secondary caries	absence	absence	absence
	Root fracture	absence	absence	absence
	Radiographic findings	normal (bone level stable, healthy periapical and periodontal tissues)	normal (bone level stable, healthy periapical and periodontal tissues)	normal (bone level stable, healthy periapical and periodontal tissues)
	Prosthetic restoration	functional	functional	functional

CAL: clinical attachment level; BoP: bleeding on probing; (+): presence; (−): absence.
